# Efficacy and safety of *Bacillus clausii* (O/C, N/R, SIN, T) probiotic combined with oral rehydration therapy (ORT) and zinc in acute diarrhea in children: a randomized, double-blind, placebo-controlled study in India

**DOI:** 10.1186/s40794-022-00166-6

**Published:** 2022-04-10

**Authors:** Keya Rani Lahiri, Raghvendra Singh, Mohini Apte, Mahantesh Patil, Amar Taksande, Rafael Varona, Godhuli Chatterjee, Manish Verma, Sandrine Brette, Marcos III Perez

**Affiliations:** 1 D Y Patil Medical College and Hospital, Nerul, Navi Mumbai, India; 2 Maulana Azad Medical College and Lok Nayak Hospital, 2 Bahadur Shah Zafar Road, New Delhi, India; 3 Government Medical College and Hospital, Medical College Square, Nagpur, India; 4 JN Medical College & KLES Dr. Prabhakar Kore Hospital & MRC, Nehru Nagar, Belgaum, India; 5 Acharya Vinoba Bhave Rural Hospital, Jawaharlal Nehru Medical College, Datta Meghe Institute of Medical Sciences, Sawangi (Meghe), Wardha, India; 6 Global Medical, Consumer Healthcare, Sanofi-Aventis, Gentilly, France; 7 Senior Medical Advisor and Clinical Study Unit Safety Lead, Clinical Study Unit (India-South East Asia Cluster), Sanofi Healthcare India Private Limited, Mumbai, Maharashtra India; 8 Sanofi Healthcare India Private Limited, Mumbai, Maharashtra India; 9 Aixial, Boulogne-Billancourt, France; 10 Global Medical, Consumer Healthcare, Sanofi-Aventis Deutschland GmbH, Frankfurt, Germany

**Keywords:** Probiotics, *Bacillus clausii*, Acute diarrhea, Childhood diarrhea, Children, Randomized, Placebo-controlled

## Abstract

**Background:**

Childhood diarrhea remains a major disease burden, particularly in developing countries, and is a leading cause of death in children aged < 5 years, worldwide. Treatment of acute diarrhea now includes probiotics to potentially reduce the duration and severity of the illness. This phase 3, randomized, placebo-controlled, double-blind study assessed the efficacy and safety of four strains (O/C, N/R, SIN, T) of *Bacillus clausii* probiotic (Enterogermina®) plus oral rehydration therapy (ORT) and zinc, versus placebo plus ORT and zinc, in infants and children in India with acute moderate diarrhea.

**Methods:**

Patients aged 6 months to 5 years with acute moderate diarrhea (WHO 2005 definition) of < 48 h’ duration were randomly assigned to receive one mini bottle of either polyantibiotic-resistant *B. clausii* (oral suspension of 2 billion spores per 5 mL bottle) or matching placebo twice daily (morning and evening) for 5 days. Exclusion criteria included known hypersensitivity to *B. clausii* or excipients in the study treatment, or to other probiotics. Patients were admitted to hospital from Day 1 and discharged ≥6 h after diarrhea resolution, or a maximum of 5 days. The primary endpoint was duration of acute diarrhea from randomization to recovery. Secondary endpoints included frequency of stools, diapers with stools, or dehydration status.

**Results:**

In total, 457 patients were randomized; 454 were treated. Similar proportions of patients showed recovery from diarrhea over the 120 h after randomization (97.0% in the *B. clausii* group [*n* = 227]; 98.0% on placebo [*n* = 227]). Median time to recovery was also similar: 42.83 (95% CI: 40.90–44.90) hours for *B. clausii* and 42.13 (95% CI: 39.80–43.87) hours for placebo. However, no statistically significant difference was observed between groups (hazard ratio = 0.93 [95% CI: 0.77–1.13]; *p* = 0.6968); nor were there statistically significant differences between groups for the secondary endpoints. Treatment with *B. clausii* was well tolerated with incidence of adverse events (9.7%) similar to that for placebo (12.3%).

**Conclusions:**

No significant difference in efficacy between *B. clausii* and placebo was demonstrated. Sample size may have been inadequate to allow detection of a between-group difference in efficacy, given the mild/moderate severity (only ~ 20% of patients had nausea/vomiting or abdominal pains) and short duration of disease among subjects, the relatively late start of treatment (most were already on Day 2 of their disease episode when study treatment started) and the effectiveness of the standard of care with ORT and zinc in both treatment groups.

**Trial registration:**

CTRI number CTRI/2018/10/016053. Registered on 17 October 2018. EudraCT number 2016-005165-31. Registered 14 May 2020 (retrospectively registered).

**Supplementary Information:**

The online version contains supplementary material available at 10.1186/s40794-022-00166-6.

## Background

While improvements in public health, sanitation and vaccination against rotavirus infection have reduced the incidence and mortality from childhood diarrhea [1–5], diarrhea nonetheless remains a major health problem, particularly in developing countries [6, 7]. A 2018 analysis of the 2016 Global Burden of Disease study estimated that diarrhea was the fifth leading cause of death among children aged < 5 years worldwide [4], while in 2017 the World Health Organization (WHO) identified it as the second leading cause of death among this age group [8]. Within India, data from 2010 placed diarrhea as the third most common cause of child mortality, responsible for an estimated 300,000 deaths per year [9]. Other consequences of diarrhea in children include malnutrition and impairment of growth and cognitive development [10]. Diarrheal diseases also impose a significant economic burden on health services, being responsible for up to a third of total pediatric admissions in India [11, 12].

Current management of acute diarrhea consists of replacement of lost fluids and electrolytes with oral rehydration therapy (ORT) [13], often with zinc supplementation; however, ORT does not reduce either the severity or the duration of diarrhea [14]. Another recommended treatment option is the use of probiotics [15], which can help nurture native gut flora and correct imbalances that may contribute to diarrheal diseases. Increased levels of beneficial bacterial flora may help form a ‘barrier’ against pathogens, through mechanisms such as competition for nutrients and gut wall receptor sites, excretion of acids and immunomodulation [16]. Several meta-analyses have suggested that the use of probiotics may improve outcomes in children with acute gastroenteritis [17–19] and some clinical guidelines have recommended their use in children with acute gastroenteritis and diarrhea [10, 20, 21].

*Bacillus clausii* is a rod-shaped, non-pathogenic, spore-forming, aerobic, gram positive bacterium [22]. In India, a *Bacillus clausii* probiotic composed of four strains of this non-pathogenic, alkali-tolerant, aerobic gram positive bacteria (O/C, N/R, SIN, T) in aqueous suspension of 2 billion colony forming units (Enterogermina®) is commercially available. The preparation retains good viability within the human gastrointestinal tract due to the spores of *B. clausii,* as with all bacteria of this genus, being highly resistant to both physical and chemical agents. Following oral administration of the preparation the spores pass unharmed through the stomach and germinate in the intestine to give rise to vegetative cells [23, 24]. *B. clausii* is currently indicated for the treatment of alterations of intestinal bacterial flora in children and adults. In children suffering from acute diarrhea, *B. clausii* has been shown to reduce rotavirus/adenovirus excretion and stool frequency [23, 25]. A systematic review of six randomized, controlled clinical trials of *B. clausii* in combination with ORT or in combination with ORT plus zinc for the treatment of acute diarrhea in almost 1300 children showed that treatment significantly reduced both the duration of diarrhea and the duration of hospitalization compared with control (ORT alone or ORT plus zinc) [26]. There was also a trend of decreasing stool frequency after *B. clausii* in combination with ORT or in combination with ORT plus zinc administration compared with the control group. The study authors concluded that *B. clausii* may represent an effective therapeutic option in acute childhood diarrhea with a good safety profile, and may aid in reducing the need for hospitalization. However, in view of the heterogeneity of the available studies, this review noted that further research was recommended. Other studies have also shown some efficacy of *B. clausii* in the treatment of diarrhea in adults [27, 28] and children [29]. The Working Group on Probiotics and Prebiotics of the European Society for Paediatric Gastroenterology, Hepatology and Nutrition (ESPGHAN) recently released a weak (conditional) recommendation [30] against *B. clausii* for acute gastroenteritis, with very low certainty of evidence. In their commentary, the Working Group highlighted the lack of high-quality data from randomized controlled trials. An expert panel of Asian physicians, including pediatricians, pediatric gastroenterologists and a pediatric infectious disease specialist all involved in the management of pediatric diarrhea, has, however, recently recommended the use of *B. clausii* spores as an adjunctive treatment with oral rehydration solution for acute viral diarrhea and stated that *B. clausii* may also be considered for the prevention of antibiotic-associated diarrhea, *Clostridium difficile*-induced diarrhea, and for the adjunctive treatment of *Helicobacter pylori* [31].

This study sought to address the unmet need for information from a well-designed, high quality, multicenter study which would provide data on the utility of *B. clausii* in the treatment of acute childhood diarrhea in India. The primary objective of this study was to investigate the efficacy of 5 days’ treatment with *B. clausii* added to ORT and zinc, compared with placebo plus ORT and zinc, in infants and children from the Indian subcontinent with acute diarrhea (less than 48 h duration).

## Methods

This phase 3, randomized, double-blind, placebo-controlled, parallel group study was conducted at nine centers across India. The study was conducted between December 2018 and March 2020 in accordance with all applicable laws, rules and regulations and with the Declaration of Helsinki and the International Council for Harmonization guidelines for Good Clinical Practice. The parents or legal guardians of all children participating in the study provided their written, informed consent at the time of the child’s enrollment. The study protocol was approved by the Independent Ethics Committee (Supplementary Appendix 1; Additional File 1) at each study site.

### Patients

Infants and children aged between 6 months and 5 years with acute moderate diarrhea (WHO 2005 definition) of less than 48 h duration were included in the study. Acute moderate diarrhea was defined as the passage of unusual loose or watery stools at least three times in the previous 24-h period, presentation with mild or moderate signs of dehydration (defined as ‘some dehydration’ by the WHO), and rehydration could be performed orally [13, 32].

Exclusion criteria included known hypersensitivity to *B. clausii* or excipients in the investigational medical product or to other probiotics, pre-existing chronic gastrointestinal disease, current presence (or history within the previous 3 months) of blood, pus or mucus in stools, persistent diarrhea (duration > 14 days), clinically significant signs or symptoms of parasitic or bacterial diarrhea, severe or persistent vomiting, severe dehydration (WHO classification) [13] or malnutrition (< 50% of average weight for age [Indian Academy of Pediatrics classification [33], or treatment with antibiotics, antiparasitics, probiotics or prebiotics (use in dairy foods was permitted but not in baby formulas), antidiarrheals, laxatives or corticosteroids (except intranasal, ophthalmic, or topical formulations) within 2 weeks prior to study enrollment. Patients with long-term use of oral or intravenous corticosteroids within 6 months of enrollment were excluded. Details of other inclusion and exclusion criteria are presented in Supplementary Appendix 2; Additional File 3.

### Study procedures and treatments

Infants and children in India with acute diarrhea were randomized in a 1:1 ratio to treatment with either the probiotic *B. clausii* preparation in combination with ORT, or matching placebo (i.e., in identical plastic vials to those containing active treatment but containing only water) plus ORT for 5 days. Zinc therapy was given to both groups for 14 days. ORT and zinc treatment are recommended by WHO and represent the current standard of care for the treatment of acute diarrhea [8, 13]. Treatment was given twice a day for 5 days i.e., Day 1 (morning) to Day 5 (evening), or Day 1 (evening) to Day 6 (morning). Patients were hospitalized from Day 1 and discharged at least 6 h after diarrhea resolution or for a maximum of 5 days corresponding to 120 h from the time to randomization. If resolution of diarrhea occurred before 5 days (120 h from the time to randomization), patients continued in the study at home until study end on Day 6. These patients were supplied with a diary for follow-up entries, for the patient’s parent or legal guardian to complete. They were required to fill in the diary relating to study treatment; and behavior and perceived efficacy scales. Stool record and food intake were also recorded. Empty vials and sachets were brought along to follow-up visits.

During the hospitalization period, efficacy and safety assessments were carried out daily by medical study staff. Stool frequency and consistency, vital signs, hydration status, and feeding patterns were assessed by the study team.

#### Treatments

One treatment kit was dispensed to each patient to cover the whole study period. Each treatment kit contained 15 mini bottles (5 mL) of either the *B. clausii* preparation or placebo (two mini bottles/day for 5 treatment days plus five mini bottles provided as a reserve [in case of vomiting or spillage]). Each mini bottle of the *B. clausii* preparation contained 2 billion spores of poly antibiotic-resistant *B. clausii* spores. To preserve study blinding, the *B. clausii* preparation and matching placebo were provided in identical 5 mL bottles labeled with a unique treatment number generated by the study sponsor. Access to treatment codes was not allowed except under circumstances that required unblinding, e.g. for reporting a suspected unexpected serious adverse reaction.

Patients swallowed the contents of two mini bottles of their assigned treatment per day, one in the morning and one in the evening, for 5 days. The doses were administered at 12 hourly intervals after correction of the dehydration, as per the protocol. The first dose was administered in the morning of Day 1 and the last (tenth) dose was administered in the evening of Day 5. If the first dose was administered in the evening of Day 1, the tenth dose was administered in the morning of Day 6. *B. clausii* or matching placebo could be taken with or without food or drink. If a patient vomited immediately after intake, or in case of spillage, a new intake could be taken but only one extra mini bottle per day was allowed, from the reserve bottles. ORT and zinc were administered after *B. clausii*/placebo, with ORT being administered for 5 days and zinc for 14 days (as recommended by the WHO). ORT (based on WHO formula) was provided in the form of sachets of 4.4 g Electral® powder containing sodium (75 mOsmol/L), potassium (20 mOsmol/L), chloride (65 mOsmol/L), citrate (10 mOsmol/L) and dextrose (75 mOsmol/L); with a total osmolarity of 245 mOsmol/L. The powder was mixed in 200 mL of boiled water or water with a low mineral content at ambient temperature to create a solution, which could be kept in a refrigerator for up to 24 h. Breastfeeding could be continued throughout rehydration. Oral zinc acetate was provided in the form of bottles containing 100 mL of ready-for-use Zinconia® syrup; this was administered as a 5 mL oral daily dose containing 20 mg elemental zinc.

Study treatment was permanently discontinued in cases of hypersensitivity reaction or severe vomiting with intravenous rehydration. For patients with worsening clinical features or requiring intravenous rehydration therapy it was advised that medical treatment was sought, and the patient was withdrawn from the study. Temporary treatment discontinuation was considered by the investigators due to suspected adverse events (AEs) or when ORT was ongoing for replacement of stool losses. Re-starting study treatment was to be done under close clinical and/or laboratory monitoring once the Investigator considered that the relationship of the event to study treatment was unlikely, and that the study selection criteria had been met. A patient’s parents/legal guardian could permanently withdraw them from study treatment at any time irrespective of the reason, or this could be the Investigator’s decision. In cases where study treatment was discontinued, the patient was to remain in the study and be assessed using the procedure normally planned for the last dosing day with study medication.

#### Assessments

Patients’ medical histories were taken at the screening/baseline visit on Day 1 (the first day of their hospitalization). At the baseline visit, all patients underwent a physical examination and assessment of vital signs, assessment of pre-duration of diarrhea prior to study entry, stool consistency according to Bristol score, food intake and breastfeeding status, stool frequency and diaper use (younger children), dehydration status and concomitant medications. Monitoring of patients for any hypersensitivity reaction such as rash was to be performed for at least 30 min after the first and second administrations of study treatment. AE monitoring, treatment compliance, and behavior and perceived efficacy scales (see below) were performed each day from study Days 1–6.

The impact of acute diarrhea on behavior was evaluated using a new observer-reported outcome (ObsRO) questionnaire developed for this study. This was to be completed at each study visit (each day) from Days 1 to 6 prior to any meaningful interaction with site staff and any physical examination. The questions were to be answered by the same parent or legal guardian approximately at the same time of each day during the entire study and be completed before the perceived efficacy scale. This behavior scale included five items to measure the caregiver’s observations of the impact of diarrhea on the child’s comfort, sleep, daily activities and eating. Items were developed based on the work of Fischbach et al. [34]. Responses were to be made using a 5-point categorical rating scale: ‘not at all’, ‘slightly’, ‘somewhat’, ‘very’, or ‘extremely’. The perceived efficacy of study treatment among caregivers was also evaluated using a new self-reported questionnaire developed for this study. The perceived efficacy scale contained a single item asking the parental caregiver “Are you worried that your child’s diarrhea is getting worse?”, the available answers being ‘not at all’ (score 1), ‘slightly’ (score 2), ‘somewhat’ (score 3), ‘very’ (score 4), or ‘extremely’ (score 5).

#### Study endpoints

The primary efficacy endpoint was the duration of acute diarrhea (in hours), as counted from the time of randomization up to recovery (the first stool recorded as normal according to Bristol Stool Scale classification (score < 5, [35]). Secondary efficacy endpoints included the frequency of stools per day, and dehydration status (WHO classification: A = no dehydration, B = some dehydration, C = severe dehydration) [13] on each day the patient was hospitalized. Frequency of stools was determined as the sum of the frequency of stools and the frequency of diapers with stools. In addition, the following exploratory endpoints were assessed including bedside stool screening for rotavirus and adenovirus (in order to evaluate the efficacy of study treatment in patients with confirmed viral diarrhea), the impact of acute diarrhea on the affected child’s behavior, the perceived efficacy of study treatment among caregivers and the time to ‘no dehydration’ and duration of acute diarrhea from first intake of study treatment.

### Statistics

Statistical analyses were generated using SAS® version 9.4.

In a previous study (EudraCT # 2014–004636-19), the Kaplan-Meier percentage of resolved diarrhea at 3 days was 76% in the *B. clausii* group and 66% in the placebo group [36]. This was translated (using exponential distribution) as a hazard ratio (HR) of 1.323, considering the diarrhea recovery as event. Based on these assumptions, a total of 401 patients with event were needed to achieve 80% power to demonstrate superiority of *B. clausii* over placebo by two-sided log-rank test at a 0.05 type I error rate. Considering a maximum diarrhea assessment at 5 days, a total of 462 patients in two arms (231 patients per arm) were anticipated to be needed in the present study to reach the targeted number of patients with event in order to determine whether there was any significant difference in the primary efficacy endpoint between the active treatment and placebo groups.

The primary efficacy analysis population was the intent-to-treat (ITT) population, defined as all randomized patients, analyzed according to the treatment group allocated by randomization. The safety population considered for safety analyses was the randomized population who received at least one dose or part of a dose of the double-blind investigational product.

Continuous data were summarized using the number of available data, mean, standard deviation (SD), median, minimum and maximum for each study conduct approach group. Categorical and ordinal data were summarized using the number and percentage of patients in each treatment group. Patients with missing data were not counted in the percentages; percentages were calculated using only available data. Statistical analyses were performed at the 5% significance level using two-sided tests or two-sided 95% confidence intervals (95% CIs). For the time-to-event outcomes, Kaplan-Meier estimates (including curves) were computed and the 95% CI for the median survival times provided. The primary endpoint, time to diarrhea recovery (in hours) was compared between the treatment groups using a stratified log-rank test through the LIFETEST procedure in SAS with the fixed factors: age (< 2 years; ≥2 years), viral status (viral; non-viral) and breastfeeding status (yes; no; mixed). HR and corresponding 95% CI were provided using a Cox proportional hazard model which included the same fixed factors as in the stratified log-rank test. Subgroup analyses were performed for patient subgroups based on the baseline characteristics of age (< 2 years; ≥2 years), viral status (viral; non-viral), and breastfeeding status (yes; no; mixed) using a similar Cox analysis approach as was used for the primary analysis, adding the corresponding subgroup factor and subgroup factor-by treatment interaction. Three sensitivity analyses – using: i) the same Cox model as in the primary analysis but without adjustment for all covariates, ii) the same Cox model as for the primary analysis but with an additional covariate, the duration of pre-inclusion (current episode) diarrhea, and iii) the same Cox model as for the primary analysis but with the intake of prohibited and/or rescue medications considered as an additional reason for censoring – were also performed. The analysis of safety endpoints was descriptive and based on the safety population. Treatment-emergent AEs (TEAEs) were defined as AEs that developed or worsened during the treatment period.

## Results

### Patient disposition and baseline characteristics

**Fig. 1:**
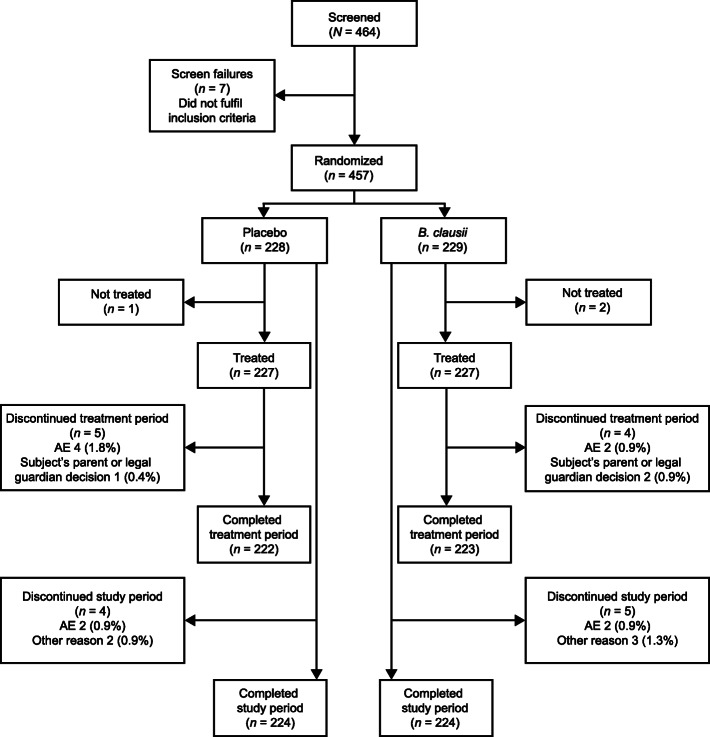
Patient disposition. AE, adverse event

Figure 1 shows the patient disposition for the study. A total of 464 patients were screened for the study, of whom 457 (*B. clausii* group *n* = 229, placebo *n* = 228; seven subjects did not meet study inclusion criteria) were randomized to study treatment (the ITT population) and 454 (99.3%) were treated (227 patients in each group) (Safety population). Three patients (0.7%) were not treated (*B. clausii* group *n* = 2, placebo group *n* = 1) due to being withdrawn from the study by their parent or guardian. In total, 448 patients (224 in each treatment group, 98.0%) completed the study. For the nine patients (2%: five patients in the *B. clausii* group, four on placebo) who did not complete the study, the reasons for discontinuation included AEs (two patients in each treatment group), and ‘other’ reasons (three patients in the *B. clausii* group and two patients on placebo).

The treatment groups were generally well balanced with regard to patients’ baseline demographic and clinical characteristics (Table 1). The mean age of the patients in each treatment group was 2 years, and the mean baseline body weight was ~ 12 kg. The mean duration of diarrhea at study entry was ~ 30 h in both treatment groups, with duration ranging from 2.9 to 48 h. Overall, 37% of patients tested positive for rotavirus and/or adenovirus at baseline. The proportion of patients who tested positive for one or both of these viruses (33% vs 41%) and for rotavirus only (18% vs 25%) was observed to be lower in the *B. clausii* versus placebo group. The mean (SD) duration between randomization and first study treatment intake was 3.9 (2.8) hours in the *B. clausii* group and 4.3 (2.6) hours in the placebo group.

**Table 1:** Patients’ baseline demographic and clinical characteristics

	*B. clausii* (*n* = 229)	Placebo (*n* = 228)	All (*n* = 457)
Age, years			
Number of subjects	229	228	457
	2.0 (1.1)	2.0 (1.1)	2.0 (1.1)
Age group, years [*n* (%)]			
Number of subjects	229	228	457
< 2	112 (48.9)	122 (53.5)	234 (51.2)
≥ 2	117 (51.1)	106 (46.5)	223 (48.8)
Sex [*n* (%)]			
Number of subjects	229	228	457
Male	113 (49.3)	112 (49.1)	225 (49.2)
Female	116 (50.7)	116 (50.9)	232 (50.8)
No. of food intakes during the day			
Number of subjects	229	228	457
	4.2 (1.8)	4.1 (1.8)	4.2 (1.8)
Breastfeeding status [*n* (%)]			
Number of subjects	229	228	457
Yes	22 (9.6)	31 (13.6)	53 (11.6)
No	111 (48.5)	108 (47.4)	219 (47.9)
Mixed	96 (41.9)	89 (39.0)	185 (40.5)
Body weight, kg			
Number of subjects	227	227	454
	12.2 (3.9)	12.0 (3.7)	12.1 (3.8)
Body weight percentile by category, kg [*n* (%)]			
Number of subjects	227	227	454
< 5th	39 (17.2)	38 (16.7)	77 (17.0)
> 5th–< 85th	119 (52.4)	119 (52.4)	238 (52.4)
> 85th–< 95th	37 (16.3)	44 (19.4)	81 (17.8)
> 95th	32 (14.1)	26 (11.5)	58 (12.8)
Body mass index, kg/m^2^			
Number of subjects	227	226	453
	17.5 (5.0)	17.4 (3.6)	17.4 (4.4)
Temperature, °C			
Number of subjects	227	227	454
	36.8 (0.4)	36.9 (0.4)	36.9 (0.4)
Systolic blood pressure, mmHg			
Number of subjects	227	226	453
	99.4 (12.0)	100.4 (12.4)	99.9 (12.2)
Diastolic blood pressure, mmHg			
Number of subjects	227	226	453
	63.5 (11.4)	63.9 (11.3)	63.7 (11.4)
Heart rate, beats/min			
Number of subjects	227	225	452
	102.6 (15.8)	101.9 (16.9)	102.2 (16.3)
Respiratory rate, breaths/min			
Number of subjects	227	227	454
	26.2 (6.4)	25.7 (6.5)	26.0 (6.5)
Medical history and disease characteristics			
Nausea and vomiting symptoms [*n* (%)]			
Number of subjects	49 (21.4)	42 (18.4)	91 (19.9)
Gastrointestinal and abdominal pains^a^ [*n* (%)]			
Number of subjects	2 (0.9)	2 (0.9)	4 (0.9)
Duration of current diarrhea^b^, hours			
Number of subjects	229	228	457
	31.1 (9.3)	29.3 (8.7)	30.2 (9.0)
No. of stools in last 24 h			
Number of subjects	229	228	457
	6.3 (4.0)	6.6 (4.3)	6.5 (4.2)
No. of stools in last 48 h^c^			
Number of subjects	229	228	457
	10.2 (5.3)	10.1 (5.3)	10.1 (5.3)
Viral status [*n* (%)]			
Number of subjects	229	228	457
Viral	76 (33.2)	94 (41.2)	170 (37.2)
Non-viral	153 (66.8)	134 (58.8)	287 (62.8)
Not valid	0	0	0
Not done	0	0	0
Rotavirus and adenovirus test status [*n* (%)]			
Number of subjects	229	228	457
Adenovirus positive	28 (12.2)	26 (11.4)	54 (11.8)
Rotavirus positive	41 (17.9)	56 (24.6)	97 (21.2)
Adenovirus and rotavirus positive	7 (3.1)	12 (5.3)	19 (4.2)
Negative	153 (66.8)	134 (58.8)	287 (62.8)
Not valid	0	0	0
Not done	0	0	0

All data in the table are expressed as mean (SD) unless otherwise stated

^a^Excluding oral and throat

^b^Duration of current diarrhea episode (hours) = (Date time of randomization – Date time of start time of current diarrhea episode)/3600

^c^Number of stools in the last 48 h includes stools in the last 24 h

### Efficacy

All efficacy analyses were performed in the ITT population.

#### Primary endpoint

**Fig. 2:**
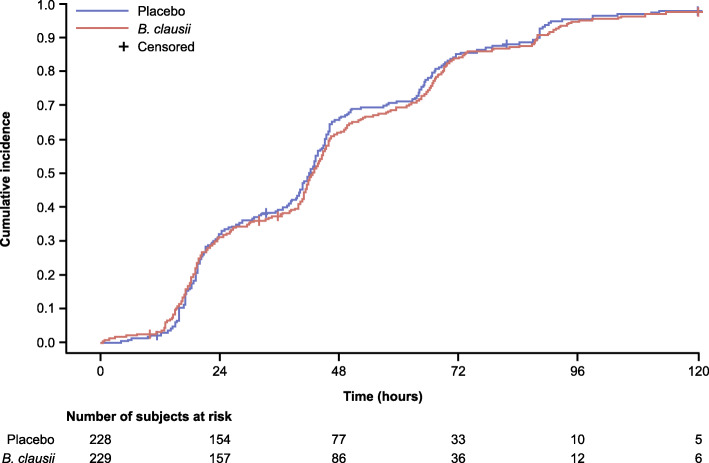
Primary endpoint: cumulative incidence and Kaplan-Meier estimate of recovery from diarrhea (ITT population). Note: Event is defined as diarrhea recovery (the first normal stool recorded according to Bristol score; a score < 5 is described as normalization of stools). The censoring patient is a patient who did not meet the event ‘recovery diarrhea’ during the 120 h after randomization ITT, intent-to-treat

Recovery from diarrhea was achieved by 97.0% (Kaplan-Meier estimate) of patients in the *B. clausii* group and 98.0% (Kaplan-Meier estimate) of those who received placebo over the 120 h after randomization. The median time to recovery was similar in the two treatment groups: 42.83 (95% CI: 40.90–44.90) hours for patients in the *B. clausii* group and 42.13 (95% CI: 39.80–43.87) hours for those in the placebo group. The incidence of recovery in the two treatment groups at each 24-h observation interval was similar and no statistically significant difference between the two treatment groups was observed (HR = 0.93 (95% CI: 0.77–1.13; *p *= 0.6968). The cumulative incidence of diarrhea recovery over time was similar (Fig. 2).

The results of the sensitivity analyses were consistent with those of the primary analyses, with no statistically significant differences between the two treatment groups. The results of subgroup analyses performed for the baseline characteristics of age (< 2 years; ≥2 years), viral status (viral [adeno/rotavirus]; non-viral) and breastfeeding status (yes; no; mixed) were also similar to the primary analyses. No treatment-by-subgroup effect was observed for any of the subgroups (See Supplementary Tables 1–3, Additional file 2).

#### Secondary efficacy endpoints

As statistical significance was not shown for the primary efficacy endpoint, no inferential analysis was performed for key secondary endpoints.

##### Frequency of stools and diapers with stools

The frequency of stools and diapers with stools was similar in the two treatment groups over the 5-day period (Table 2). The total mean (SD) number of stools and diapers with stool in the *B. clausii* group was 4.56 (2.25) on Day 1 and 1.66 (1.67) on Day 5, respectively, versus 4.91 (2.39) and 1.63 (1.15) in the placebo group.

**Table 2:** Frequency of stool and diapers with stool by visit (ITT population)

	*B. clausii* (*n* = 229)	Placebo (*n* = 228)
No. of unusual stools within 48 h of visit 1		
Number	229	228
Mean [SD]	10.2 [5.3]	10.1 [5.3]
0 times	0	0
1 time	0	0
2 times	0	0
3 times	0	2 (0.9)
> 3 times	229 (100.0)	226 (99.1)
No. of unusual stools within 24 h of visit 1		
Number	229	228
Mean [SD]	6.3 [4.0]	6.6 [4.3]
0 times	0	0
1 time	0	0
2 times	0	0
3 times	30 (13.1)	26 (11.4)
> 3 times	199 (86.9)	202 (88.6)
Day 1		
Total no. of stools and diapers with stool		
Number	229	228
Mean [SD]	4.6 [2.3]	4.9 [2.4]
0 times	1 (0.4)	2 (0.9)
1 time	2 (0.9)	2 (0.9)
2 times	37 (16.2)	28 (12.3)
3 times	37 (16.2)	35 (15.4)
> 3 times	152 (66.4)	161 (70.6)
Day 2		
Total no. of stools and diapers with stool		
Number	228	227
Mean [SD]	3.8 [2.5]	3.8 [2.3]
0 times	2 (0.9)	1 (0.4)
1 time	19 (8.3)	24 (10.6)
2 times	49 (21.5)	46 (20.3)
3 times	68 (29.8)	57 (25.1)
> 3 times	90 (39.5)	99 (43.6)
Day 3		
Total no. of stools and diapers with stool		
Number	225	226
Mean [SD]	2.4 [2.0]	2.5 [1.8]
0 times	3 (1.3)	4 (1.8)
1 time	79 (35.1)	75 (33.2)
2 times	70 (31.1)	62 (27.4)
3 times	31 (13.8)	44 (19.5)
> 3 times	42 (18.7)	41 (18.1)
Day 4		
Total no. of stools and diapers with stool		
Number	211	214
Mean [SD]	2.0 [2.1]	2.0 [1.8]
0 times	2 (0.9)	2 (0.9)
1 time	112 (53.1)	111 (51.9)
2 times	55 (26.1)	57 (26.6)
3 times	21 (10.0)	24 (11.2)
> 3 times	21 (10.0)	20 (9.3)
Day 5		
Total no. of stools and diapers with stool		
Number	200	201
Mean [SD]	1.7 [1.7]	1.6 [1.2]
0 times	2 (1.0)	2 (1.0)
1 time	135 (67.5)	121 (60.2)
2 times	41 (20.5)	51 (25.4)
3 times	10 (5.0)	15 (7.5)
> 3 times	12 (6.0)	12 (6.0)

Data expressed as Mean [SD] and as n (%) for number of times

Note: Data on unusual stools within the 24 and 48 h of visit 1 date is coming from oral information done by parents/legal guardians

ITT, intent-to-treat

##### Dehydration status

**Fig. 3:**
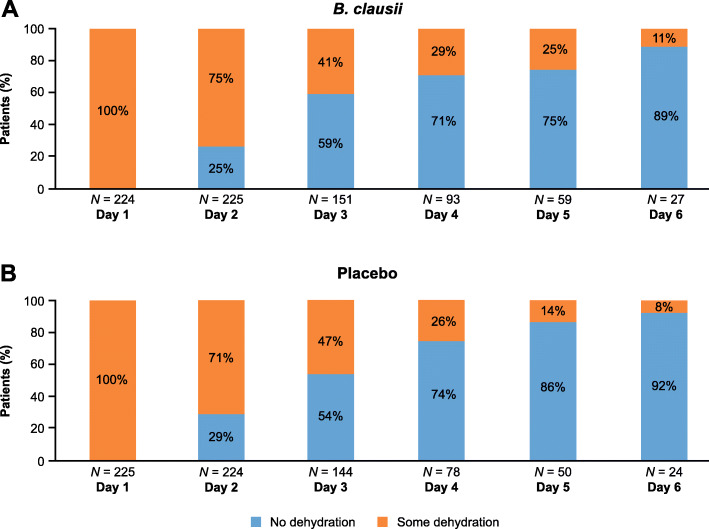
Patients’ dehydration status during hospitalization following treatment with A) *B. clausii* and B) placebo. *Note:* One patient (0.4%) treated with placebo had severe dehydration on Day 2

The evaluation of dehydration as per WHO classification prior to first study treatment intake and on each study day before each study drug intake during hospitalization showed that recovery from dehydration was similar in both treatment groups. All patients had some dehydration on Day 1 (5–10% fluid deficit as percentage of body weight), but by Day 5 most patients (74.6% of patients in the *B. clausii* group, 86.0% of those on placebo) evaluated had no dehydration (< 5% fluid deficit as percentage of body weight) (Fig. 3).

#### Exploratory efficacy endpoints

##### Bedside stool screening for rotavirus and adenovirus

In total, 76 (33.2%) patients in the *B. clausii* group and 94 (41.2%) patients in the placebo group were adeno/rotavirus-positive. Rotavirus was the more prevalent of the two viruses, being identified in 41 (17.9%) patients in the *B. clausii* group and 56 (24.6%) patients in the placebo group. A total of 28 (12.2%) patients in the *B. clausii* group and 26 (11.4%) in the placebo group were adenovirus-positive. The efficacy of study treatment in patients with confirmed viral diarrhea was evaluated through the primary endpoint subgroup analysis. No treatment effect was observed (see Supplementary Table 2, Additional file 2).

##### Impact of acute diarrhea on child behavior

**Fig. 4:**
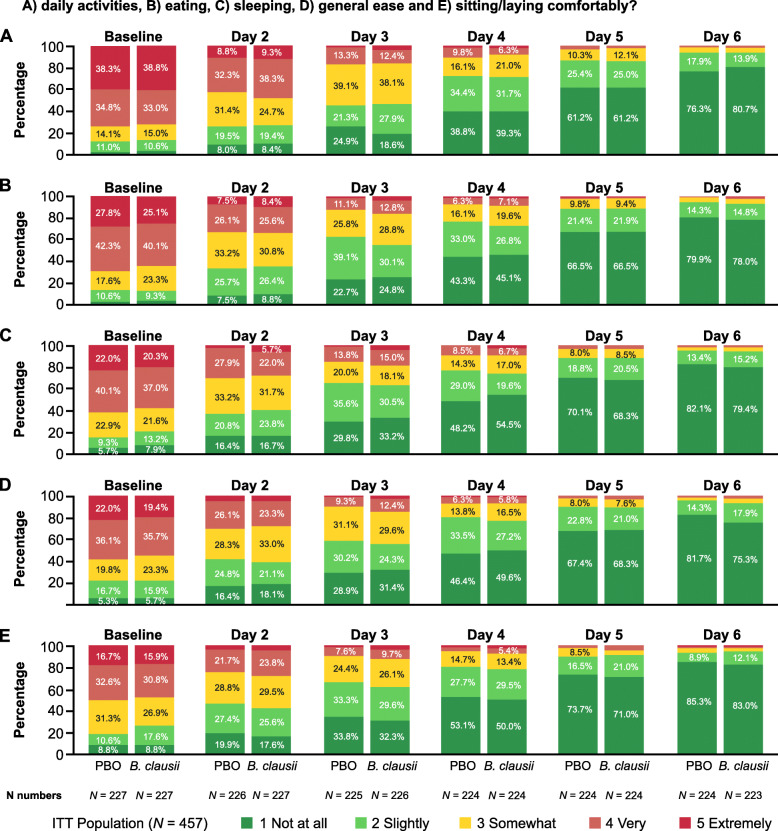
Impact of diarrhea on aspects of affected children’s behavior. A) daily activities, B) eating, C) sleeping, D) general ease and E) sitting/laying comfortably?. ITT, intent-to-treat; PBO, placebo

Most caregivers considered that their child’s behavior improved over the study period, with the responses similar in the two treatment groups (Fig. 4). Across the five parameters assessed (daily activities, eating, sleep, wincing/crying/complaining/squirming, sitting or lying comfortably) at baseline most caregivers responded in the categories ‘very likely’ (*B. clausii* group 30.8–40.1%, placebo group 32.6–42.3%) or ‘extremely likely’ (15.9–38.8%, 16.7–38.3%) that diarrhea disturbed their child’s behavior. In contrast, by Day 5 or 6 most responded that it affected their child’s behavior ‘slightly’ (Day 5: *B. clausii* group 20.5–25.0%, placebo group 16.5–25.4%; Day 6: 12.1–17.9%, 8.9–17.9%) or ‘not at all’ (Day 5: *B. clausii* group 61.2–71.0%, placebo group 61.2–73.7%; Day 6: 75.3–83.0%, 76.3–85.3%). For all analyses of the impact of acute diarrhea on child behavior the responses were similar in the two treatment groups.

##### Perceived efficacy of treatment among caregivers

**Fig. 5:**
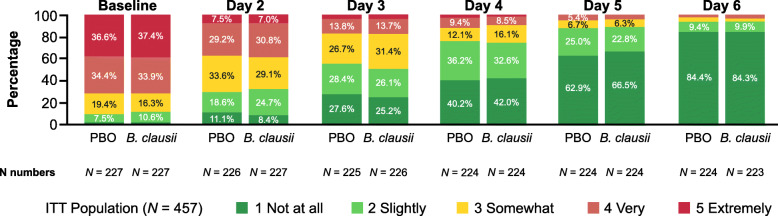
Perceived efficacy of study treatments among caregivers following the question “Are you worried that your child’s diarrhea is getting worse?”. ITT, intent-to-treat; PBO, placebo

The caregiver’s perceptions of their child’s diarrhea improved over time, with the responses similar in the two treatment groups (Fig. 5). At baseline, most caregivers perceived that their child’s diarrhea was getting worse, but by Day 6, 84% of caregivers in each treatment group responded ‘not at all’ in the perceived efficacy of treatment questionnaire. For all analyses of the perceived efficacy of treatment among the patient caregivers, the responses were similar in the two treatment groups.

##### Time to first ‘no dehydration’ status

The first ‘no dehydration’ status was achieved by 64.0% of patients (Kaplan-Meier estimate) in the *B. clausii* group and 65.0% (Kaplan-Meier estimate) in the placebo group over the 120 h after first dose of study treatment. The median time to first ‘no dehydration’ status in the two treatment groups was the same: 48.00 (95% CI: 38.48–67.67) hours in the *B. clausii* group and 48.00 (95% CI: 37.17–63.50) hours in the placebo group. There was no significant difference between the two treatment groups: HR = 1.00 (95% CI: 0.79–1.26). The cumulative incidence of first ‘no dehydration’ status over time was also similar in the two treatment groups.

##### Recovery from diarrhea after the start of study treatment

Recovery from diarrhea was similar in the two treatment groups over the 120 h after starting study treatment, and was reported in 97.0% (Kaplan-Meier estimate) of patients in the *B. clausii* group and 98.0% of those on placebo. The overall median time to recovery from first intake of study treatment was similar in the two treatment groups: 37.70 (95% CI: 36.47–40.00) hours in the *B. clausii* group versus 36.93 (95% CI: 33.75–39.18) hours in the placebo group. There was no significant difference between the two treatment groups: HR = 0.93 (95% CI: 0.77–1.13). The cumulative incidence of recovery from diarrhea over time was similar in the two groups.

### Safety and tolerability

The mean duration of study treatment was 131.0 (SD 10.9) hours in the *B. clausii* group and 130.6 (14.6) hours in the placebo group. Most patients in each treatment group (98.2% *B. clausii*, 97.8% placebo) received study treatment for > 120 h, even if at home after hospital discharge. Treatment compliance was above 80% in 96.9% of patients in the *B. clausii* group and 96.5% of those on placebo.

The incidences of TEAEs were similar between the two treatment groups (Table 3). In total, 22 (9.7%) patients in the *B. clausii* group and 28 (12.3%) in the placebo group experienced AEs. The most frequently reported events were vomiting (2.6% patients in each treatment group), pyrexia (2.2% for *B. clausii* vs 2.6% for placebo), and nasopharyngitis (2.2% patients vs 1.3%). Most patients with AEs experienced events of mild severity (*B. clausii* group 19 [8.4%] patients, placebo group 26 [11.5%] patients). Moderate vomiting and pyrexia were each reported in one patient, respectively, in the *B. clausii* group. Two patients (0.9%) in each treatment group experienced severe dehydration, with all except one patient in the *B. clausii* group discontinuing study treatment. No deaths, serious AEs, AEs of special interest (AESI) or AEs that were considered by the investigator to be possibly related to study treatment were reported during the study.

**Table 3:** TEAEs by System Organ Class and Preferred Term^a^

	*B. clausii* (*n *= 227)	Placebo (*n *= 227)
Patients with any TEAE	22 (9.7)	28 (12.3)
Patients with any treatment-emergent serious AE	0	0
Patients with any treatment-emergent AESI	0	0
Patients with any TEAE leading to death	0	0
Patients with any TEAE leading to definitive		
treatment discontinuation	2 (0.9)	3 (1.3)
Infections and infestations	6 (2.6)	7 (3.1)
Nasopharyngitis	5 (2.2)	3 (1.3)
Upper respiratory tract infection	1 (0.4)	3 (1.3)
Dysentery	0	1 (0.4)
Blood and lymphatic system disorders	1 (0.4)	0
Anemia	1 (0.4)	0
Metabolism and nutrition disorders	3 (1.3)	3 (1.3)
Dehydration	2 (0.9)	2 (0.9)
Hypokalemia	2 (0.9)	0
Lactose intolerance	0	1 (0.4)
Eye disorders	0	1 (0.4)
Periorbital swelling	0	1. (0.4)
Respiratory, thoracic and mediastinal disorders	1 (0.4)	4 (1.8)
Rhinorrhea	1 (0.4)	2 (0.9)
Cough	0	2 (0.9)
Gastrointestinal disorders	6 (2.6)	8 (3.5)
Vomiting	6 (2.6)	6 (2.6)
Upper abdominal pain	0	1 (0.4)
Diarrhea	0	1 (0.4)
Skin and subcutaneous tissue disorders	2 (0.9)	0
Rash	1 (0.4)	0
Erythematous rash	1 (0.4)	0
General disorders and administration site conditions	5 (2.2)	6 (2.6)
Pyrexia	5 (2.2)	6 (2.6)

^a^MedDRA dictionary 23.0

Data expressed as n (%)

AESI, adverse event of special interest; TEAE, treatment-emergent adverse event

Five patients experienced TEAEs leading to discontinuation of study treatment (2 [0.9%] in the *B. clausii* group and three [1.3%] patients in the placebo group). These comprised three cases of severe dehydration (affecting one [0.4%] patient in the *B. clausii* group and two [0.9%] patients in the placebo group), mild dysentery (one [0.4%] patient in the placebo group) and mild erythematous rash (one [0.4%] patient in the *B. clausii* group). None of these events were considered to be serious or related to study treatment by the investigator. The patients with severe dehydration were treated with intravenous rehydration as rescue medication, which was a reason for discontinuation as per-study protocol. One further patient in the placebo group was discontinued from study treatment due to acute kidney injury, hypokalemia, and hyponatremia with onset prior to administration of the first placebo dose.

## Discussion

In the primary efficacy endpoint analysis of the duration of acute diarrhea from the time of randomization, diarrhea recovery was similar in the two treatment groups. Over the 120-h period after randomization, diarrhea recovery was achieved by 97.0% (Kaplan-Meier estimate) of patients in the *B. clausii* group and 98.0% of patients in the placebo group. The median time to recovery (42.8 vs 42.1 h) and cumulative incidence of diarrhea recovery over time were also similar in the two treatment groups. With regard to secondary endpoints, a more than two-fold decrease in the frequency of stools was observed in both treatment groups over the 5-day treatment period, and no treatment effect was observed. Recovery from dehydration was also similar in the two treatment groups. While all patients had ‘some dehydration’ on Day 1, by Day 5, 75% of patients in the *B. clausii* group and 86.0% of those on placebo (not significant) had ‘no dehydration’.

The results of the sensitivity analyses and of the subgroup analyses performed for baseline characteristics of age (< 2 years; ≥2 years), viral status (viral; non-viral) and breastfeeding status (yes; no; mixed) were similar to the primary analyses, showing no significant difference between the *B. clausii* and placebo groups.

A number of exploratory endpoints were also assessed including the potential impact of rota−/adenovirus status on treatment efficacy. Rotavirus was found to be present in 21% of patients in the *B. clausii* group and 30% of those who received placebo, while just over 15% of patients in each group were adenovirus positive. This is broadly consistent with the findings of a study of virus prevalence in Indian children with acute gastroenteritis (conducted before the introduction of rotavirus vaccination) which detected rotavirus in 18% of subjects and adenovirus in 10% (with astrovirus detected in 13% and coinfections in 11%) [37]. No difference in treatment efficacy according to baseline viral (adenovirus/rotavirus) status was observed. In the exploratory analyses to evaluate the impact of acute diarrhea on children’s behavior and of the perceived efficacy of treatment among caregivers, improvements were seen in both treatment groups over the study period. The responses in these analyses were similar in the two treatment groups and followed a similar pattern to that for diarrhea recovery. Finally, the exploratory analysis of time to first ‘no dehydration’ status was achieved by a similar proportion of patients in the two treatment groups (~ 65%) (Kaplan-Meier estimate) over the 120 h after first dose of study treatment; the median time to first ‘no dehydration’ status in the two treatment groups was the same (48 h).

With regard to safety, in the present study treatment with *B. clausii* was very well tolerated and there were no unexpected safety findings. The type and incidence of AEs observed were similar in the *B. clausii* and placebo groups and no deaths, serious AEs, treatment-related AEs or AESI were reported. Treatment with *B. clausii* was also shown to be well tolerated in the large, open-label CODDLE study in over 3,000 Filipino children with acute diarrhea, with an AE rate of just 0.09% [38].

A number of possible reasons why this study did not demonstrate a significant difference in efficacy with *B. clausii* compared with placebo can be posited. Firstly, at least partly due to the strict exclusion criteria applied, the study population generally had moderate or even mild disease, according to WHO classification [13]. Only around 20% of patients had nausea/vomiting or abdominal pains, making it more difficult to discern differences in study treatment efficacy. The disease course was also quite short in most patients and most of them were already on Day 2 of their disease episode on enrollment (the mean duration of diarrhea at study entry was ~ 30 h in both treatment groups), allowing little scope for demonstration of efficacy of the probiotic treatment. Another potentially important factor mitigating against the detection of a difference in study treatment efficacy may have been the effectiveness of the standard of care with ORT and zinc in both groups. The short duration of disease in our study population might, to varying degrees, reflect the effectiveness of the ORT/zinc treatment given to all patients, the observed high degree of compliance with study treatments and the effect of community rotavirus immunization programs. The effects of rotavirus immunization programs to cause a shift in the etiopathogenesis of acute gastroenteritis away from rotavirus to norovirus and other pathogens (as has been found in the USA [39, 40]) might offer an explanation as to why other recent studies have failed to demonstrate clinical benefits from the use of probiotics in children with acute gastroenteritis [41, 42] but in this regard it should be noted that another recent study by Freedman et al. which identified pathogens from patients’ stool samples failed to demonstrate any virus-specific benefits of probiotic treatment with *Lactobacillus rhamnosus* and *L. helveticus* [43].

Whatever the reason(s) why recent randomized trials have not demonstrated a clear benefit for the use of probiotics in acute gastroenteritis in children (reflected by the fact that the recent ESPGHAN guidelines make only weak recommendations [30] for the use of some probiotics [44]), it is clear that further, carefully designed clinical trials are required to better determine the utility of this therapy modality. A growing body of data from studies such as our own and future trials will provide a better understanding of the therapeutic utility of probiotics in the treatment of diarrhea and allow for more reliable, robust evidence-based decision-making. Our own study indicates that a large study population might be necessary to help identify potentially small differences in outcome between treatment groups. In this regard, a large, multinational study could provide both the benefit of a large patient population and might also help us to detect any differences in probiotic efficacy that might relate to differing healthcare and societal environments (rotavirus vaccination, use of zinc, nutrition status, socioeconomic status etc.). The design of these trials might also incorporate measures to broaden the profile of children recruited by, for example, including children with fever and those presenting with symptoms of new onset acute gastroenteritis, and other measures to promote the early enrollment of study subjects (for example by excluding patients with diarrhea onset ≥48 h prior to enrollment) to try to ensure that study treatment is not started too late in the disease course to allow for the detection of smaller between-group efficacy differences. Future trials might also consider the inclusion of patients receiving antibiotics or those with non-viral/bacterial gastroenteritis and, while the recent study by Freedman et al. [43] did not demonstrate any virus-specific benefits of probiotic treatment, the inclusion of PCR testing of patients’ stools would help us to determine whether probiotics might differ in their efficacy according to the pathogen involved. Trials of higher doses and/or longer durations of probiotic treatment that might better reflect ‘real world’ usage of probiotics might also be considered. The inclusion of study endpoints such as total duration of diarrhea, diarrhea severity scores, hospitalization rates, number of school days or parent/guardian workdays lost (and assessment by socioeconomic status, e.g., household income) might also help future trials determine more holistically the benefits or otherwise of probiotic treatment.

## Conclusions

This study was not able to demonstrate a significant clinical benefit versus placebo for the use of *B. clausii* added to the recommended therapy for the treatment of acute diarrhea in Indian children. Due to a combination of short disease duration, the relatively late start of study treatment, and the effectiveness of the ORT/zinc treatment that all patients received, the sample proved insufficient to enable discernment of a between-group difference in efficacy. *B. clausii* has previously demonstrated efficacy in reducing stool frequency and disease duration in children with acute diarrhea [26] and has a well-documented good safety and tolerability profile [38]. In view of the previous encouraging study results with this probiotic preparation, large multicenter studies incorporating lessons learned from this and other trials should be conducted to better understand the potential utility of *B. clausii* in viral, non-viral and antibiotic-associated diarrhea in children. Community-based studies of preventive efficacy among children in deprived urban settings may also provide valuable public health insight.

## Supplementary Information

**Additional file 1: Supplementary Appendix 1.** *Independent Ethics Committees*. IEC: Independent Ethics Committee

**Additional file 2: Supplementary Table 1.** Subgroup analysis of diarrhea recovery according to baseline characteristics: age (< 2 years; ≥2 years). * Only for descriptive purposes. Note: Interaction test from the Cox proportional hazard model including the factor, treatment effect and the treatment by factor interaction. Cox model was performed using the Phreg procedure in SAS. The method employed to handle ties is Efron and the 95% CI is computed using Wald. Kaplan-Meier method was used to estimate the cumulative incidence. CI, confidence interval; HR, hazard ratio**. Supplementary Table 2**. Subgroup analysis of diarrhea recovery according to baseline characteristics: viral status (viral; non-viral). * Only for descriptive purposes. Note: Interaction test from the Cox proportional hazard model including the factor, treatment effect and the treatment by factor interaction. Cox model was performed using the Phreg procedure in SAS. The method employed to handle ties is Efron and the 95% CI is computed using Wald. Kaplan-Meier method was used to estimate the cumulative incidence. CI, confidence interval; HR, hazard ratio . **Supplementary Table 3.** Subgroup analysis of diarrhea recovery according to baseline characteristics: breastfeeding (no breastfeeding; breastfeeding alone; mixed breastfeeding). * Only for descriptive purposes. Note: Interaction test from the Cox proportional hazard model including the factor, treatment effect and the treatment by factor interaction. Cox model was performed using the Phreg procedure in SAS. The method employed to handle ties is Efron and the 95% CI is computed using Wald. Kaplan-Meier method was used to estimate the cumulative incidence. CI, confidence interval; HR, hazard ratio

**Additional file 3: Supplementary Appendix 2.** *Additional Exclusion Criteria.* In addition to those listed in the main study paper, additional exclusion criteria included critical illness, chronic diseases of the endocrine, cardiovascular, renal, or respiratory system (or any other clinically significant condition that might jeopardize a patient’s condition or study outcomes in the view of the Investigator), a history of or current presence of conditions known to produce immunodeficiency (congenital or acquired immunodeficiency syndromes, immunosuppressant therapy), presence of an in-dwelling vascular access line, a history of or current pancreatitis, history of abdominal surgery, bilious emesis, or participation in another clinical trial within the past 3 months

## Data Availability

Qualified researchers may request access to patient level data and related study documents including the clinical study report, study protocol with any amendments, blank case report form, statistical analysis plan, and dataset specifications. Patient level data will be anonymized and study documents will be redacted to protect the privacy of our trial participants. Further details on Sanofi’s data sharing criteria, eligible studies, and process for requesting access can be found at: https://www.clinicalstudydatarequest.com/.

## Abbreviations

AE: adverse event
AESI: adverse event of special interest
CI: confidence interval
ESPGHAN: European Society for Paediatric Gastroenterology, Hepatology and Nutrition
HR: hazard ratio
ITT: intent-to-treat
ObsRO: observer-reported outcome
ORT: oral rehydration therapy
SD: standard deviation
TEAE: treatment-emergent adverse event
WHO: World Health Organization
