# Supplementary material for: Efficacy and safety of Bacillus clausii (O/C, N/R, SIN, T) probiotic combined with oral rehydration therapy (ORT) and zinc in acute diarrhea in children: a randomized, double-blind, placebo-controlled study in India
Source: Trop Dis Travel Med Vaccines. 2022 Apr 10;8:9. doi: 10.1186/s40794-022-00166-6 (PMC8994895; doi:10.1186/s40794-022-00166-6)
Supplement: Supplementary file 1 — Additional file 1: Supplementary Appendix 1. Independent Ethics Committees. IEC: Independent Ethics Committee [file 40794_2022_166_MOESM1_ESM.docx]

**Supplementary Appendix 1.**

***Independent Ethics Committees***

| **Study Center No.** | **Local IEC** |
| --- | --- |
| 3560001 | Ethics Committee Institute of Child Health, 1,  Dr Biresh Guha Street, Kolkata 700017, India |
| 3560003 | Ethics Committee, Dr. D. Y. Patil Vidyapeeth-  Pune, Sant Tukaram Nagar, Pimpri, Pune Maharashtra - 411018, India |
| 3560005 | Institutional Ethics Committee  D Y Patil Medical College and Hospital, Plot  No. 2, Sector 5, Nerul, Navi Mumbai- 400706, India |
| 3560007 | Institutional Ethics Committee-Cloudnine  Hospital, Jayanagar, Bengaluru 560011, Karnataka, India |
| 3560010 | Institutional Ethics Committee, Maulana Azad, Medical College, Lok Nayak Hospital, Bahadur Shah Zafar, Marg, New Delhi - 110002, India |
| 3560015 | Ethics Committee-M. S. Ramaiah Medical College and Hospitals, Bangalore 560054, Karnataka, India |
| 3560019 | Institutional Ethics Committee-Datta Meghe  Institute of Medical Sciences, Sawangi  (Meghe) , Wardha – 442004 Maharashtra, India |
| 3560020 | Institutional Ethics Committee – KLE  University, Nehru Nagar, Belgaum – 590010, India |
| 3560021 | Institutional Ethics Committee-Government  Medical College, Nagpur, 440003, Maharashtra, India |

IEC: Independent Ethics Committee
