# Supplementary material for: Efficacy and safety of Bacillus clausii (O/C, N/R, SIN, T) probiotic combined with oral rehydration therapy (ORT) and zinc in acute diarrhea in children: a randomized, double-blind, placebo-controlled study in India
Source: Trop Dis Travel Med Vaccines. 2022 Apr 10;8:9. doi: 10.1186/s40794-022-00166-6 (PMC8994895; doi:10.1186/s40794-022-00166-6)
Supplement: Supplementary file 2 — Additional file 2: Supplementary Table 1. Subgroup analysis of diarrhea recovery according to baseline characteristics: age (< 2 years; ≥2 years). * Only for descriptive purposes. Note: Interaction test from the Cox proportional hazard model including the factor, treatment effect and the treatment by factor interaction. Cox model was performed using the Phreg procedure in SAS. The method employed to handle ties is Efron and the 95% CI is computed using Wald. Kaplan-Meier method was used to estimate the cumulative incidence. CI, confidence interval; HR, hazard ratio. Supplementary Table 2. Subgroup analysis of diarrhea recovery according to baseline characteristics: viral status (viral; non-viral). * Only for descriptive purposes. Note: Interaction test from the Cox proportional hazard model including the factor, treatment effect and the treatment by factor interaction. Cox model was performed using the Phreg procedure in SAS. The method employed to handle ties is Efron and the 95% CI is computed using Wald. Kaplan-Meier method was used to estimate the cumulative incidence. CI, confidence interval; HR, hazard ratio . Supplementary Table 3. Subgroup analysis of diarrhea recovery according to baseline characteristics: breastfeeding (no breastfeeding; breastfeeding alone; mixed breastfeeding). * Only for descriptive purposes. Note: Interaction test from the Cox proportional hazard model including the factor, treatment effect and the treatment by factor interaction. Cox model was performed using the Phreg procedure in SAS. The method employed to handle ties is Efron and the 95% CI is computed using Wald. Kaplan-Meier method was used to estimate the cumulative incidence. CI, confidence interval; HR, hazard ratio [file 40794_2022_166_MOESM2_ESM.docx]

**Supplementary Tables**

**Supplementary Table 1** Subgroup analysis of diarrhea recovery according to baseline characteristics: age (<2 years; ≥2 years)

|  | **Placebo** | ***B. clausii*** | ***p*-value interaction*** |
| --- | --- | --- | --- |
| Age  <2 years  Number assessed  Number censored  Number of events  Cumulative incidence at 120 hours  HR  95% CI  ≥2 years  Number assessed  Number censored  Number of events  Cumulative incidence at 120 hours  HR  95% CI | 122  8  114  0.96  106  0  106  1.00 | 112  6  106  0.95  0.92  0.70–1.19  117  3  114  0.99  0.96  0.74–1.25 | 0.800 |

* Only for descriptive purposes.

Note: Interaction test from the Cox proportional hazard model including the factor, treatment effect and the treatment by factor interaction. Cox model was performed using the Phreg procedure in SAS. The method employed to handle ties is Efron and the 95% CI is computed using Wald. Kaplan-Meier method was used to estimate the cumulative incidence

CI, confidence interval; HR, hazard ratio

**Supplementary Table 2** Subgroup analysis of diarrhea recovery according to baseline characteristics: viral status (viral; non-viral)

|  | **Placebo** | ***B. clausii*** | ***p*-value interaction*** |
| --- | --- | --- | --- |
| Vital status  Non-viral  Number assessed  Number censored  Number of events  Cumulative incidence at 120 hours  HR  95% CI  Viral  Number assessed  Number censored  Number of events  Cumulative incidence at 120 hours  HR  95% CI | 134  6  128  0.97  94  2  92  0.99 | 153  8  145  0.96  0.91  0.71–1.15  76  1  75  1.00  1.00  0.74–1.36 | 0.603 |

* Only for descriptive purposes.

Note: Interaction test from the Cox proportional hazard model including the factor, treatment effect and the treatment by factor interaction. Cox model was performed using the Phreg procedure in SAS. The method employed to handle ties is Efron and the 95% CI is computed using Wald. Kaplan-Meier method was used to estimate the cumulative incidence

CI, confidence interval; HR, hazard ratio

**Supplementary Table 3** Subgroup analysis of diarrhea recovery according to baseline characteristics: breastfeeding (no breastfeeding; breastfeeding alone; mixed breastfeeding)

|  | **Placebo** | ***B. clausii*** | ***p*-value interaction*** |
| --- | --- | --- | --- |
| Breastfeeding status  No  Number assessed  Number censored  Number of events  Cumulative incidence at 120 hours  HR  95% CI  Yes  Number assessed  Number censored  Number of events  Cumulative incidence at 120 hours  HR  95% CI  Mixed  Number assessed  Number censored  Number of events  Cumulative incidence at 120 hours  HR  95% CI | 108  0  108  1.00  31  3  28  0.93  89  5  84  0.96 | 111  5  106  0.97  0.88  0.67–1.15  22  1  21  0.95  0.83  0.47–1.47  96  3  93  0.98  1.07  0.79–1.43 | 0.561 |

* Only for descriptive purposes.

Note: Interaction test from the Cox proportional hazard model including the factor, treatment effect and the treatment by factor interaction. Cox model was performed using the Phreg procedure in SAS. The method employed to handle ties is Efron and the 95% CI is computed using Wald. Kaplan-Meier method was used to estimate the cumulative incidence

CI, confidence interval; HR, hazard ratio
