# Supplementary material for: Efficacy and safety of Bacillus clausii (O/C, N/R, SIN, T) probiotic combined with oral rehydration therapy (ORT) and zinc in acute diarrhea in children: a randomized, double-blind, placebo-controlled study in India
Source: Trop Dis Travel Med Vaccines. 2022 Apr 10;8:9. doi: 10.1186/s40794-022-00166-6 (PMC8994895; doi:10.1186/s40794-022-00166-6)
Supplement: Supplementary file 3 — Additional file 3: Supplementary Appendix 2. Additional Exclusion Criteria. In addition to those listed in the main study paper, additional exclusion criteria included critical illness, chronic diseases of the endocrine, cardiovascular, renal, or respiratory system (or any other clinically significant condition that might jeopardize a patient’s condition or study outcomes in the view of the Investigator), a history of or current presence of conditions known to produce immunodeficiency (congenital or acquired immunodeficiency syndromes, immunosuppressant therapy), presence of an in-dwelling vascular access line, a history of or current pancreatitis, history of abdominal surgery, bilious emesis, or participation in another clinical trial within the past 3 months [file 40794_2022_166_MOESM3_ESM.docx]

**Supplementary Appendix 2.**

***Additional Exclusion Criteria***

In addition to those listed in the main study paper, additional exclusion criteria included critical illness, chronic diseases of the endocrine, cardiovascular, renal, or respiratory system (or any other clinically significant condition that might jeopardize a patient’s condition or study outcomes in the view of the Investigator), a history of or current presence of conditions known to produce immunodeficiency (congenital or acquired immunodeficiency syndromes, immunosuppressant therapy), presence of an in-dwelling vascular access line, a history of or current pancreatitis, history of abdominal surgery, bilious emesis, or participation in another clinical trial within the past 3 months.
